# Prognostic utility of oral neutrophil counts in high‐risk periodontitis: A retrospective study

**DOI:** 10.1002/jper.11360

**Published:** 2025-07-08

**Authors:** Braedan R. J. Prete, Abdelahhad Barbour, Chunxiang Sun, Howard C. Tenenbaum, Michael B. Goldberg, Michael Glogauer

**Affiliations:** ^1^ Faculty of Dentistry University of Toronto Toronto Ontario Canada; ^2^ Department of Dentistry, Centre for Advanced Dental Research and Care Mount Sinai Hospital Toronto Ontario Canada; ^3^ Department of Dental Oncology, Maxillofacial and Ocular Prosthetics Princess Margaret Cancer Centre Toronto Ontario Canada

**Keywords:** bacteria, host–microbiota interaction, inflammation, neutrophil(s), periodontal diseases

## Abstract

**Background:**

Oral polymorphonuclear neutrophil (oPMN) levels are potential biomarkers for differentiating between stages and grades of periodontitis. We compared the diagnostic utility of oPMN levels with bleeding on probing percentage (BOP%) and microbial load in high‐risk patients with periodontitis.

**Methods:**

Sixty‐two subjects were divided into four categories based on periodontitis stage and grade: stage II periodontitis (S2P, *n* = 19), stage III periodontitis (S3P, *n* = 43), grade B periodontitis (GBP, *n* = 34), and grade C periodontitis (GCP, *n* = 28). Clinical parameters included probing depth (PD), BOP%, and clinical attachment loss (CAL). Associations between oPMN levels and BOP% were compared to periodontitis stage and grade, and the presence of the periodontal pathogens *Porphyromonas gingivalis* (*Pg*), *Treponema denticola* (*Td*), *Tannerella forsythia* (*Tf*), *Aggregatibacter actinomycetemcomitans*, and *Prevotella intermedia* (*Pi*) in the gingivocrevicular fluid (GCF).

**Results:**

Both oPMN levels and BOP% were associated with increasing stage and grade of periodontitis; however, better sensitivity, specificity, and predictive values for differentiating between GBP versus GCP were observed with oPMN. Significant positive associations were found between oPMN level and the detection of *Pg* and *Pi*.

**Conclusions:**

OPMN level can be used to differentiate between grade B and C periodontitis. Likewise, the presence of periodontal pathogens *Pg* and *Pi* correlated with the oPMN level. Given these findings, oPMN level may be useful as a multipurpose clinical biomarker in terms of diagnosing periodontitis and determining the risk of disease progression.

**Plain language summary:**

Periodontitis is a serious gum disease that can lead to tooth loss and is linked to other health issues. Currently, bleeding of the gums after probing is one method used to assess the disease activity, but this method is not always accurate. In this study, we investigated whether counting a type of immune cell called oral neutrophil found in saliva could provide a better way to detect and measure the severity of periodontitis. We examined 62 patients with different stages and grades of the disease. We found that the number of oral neutrophils was a better tool for identifying more severe cases and those at a higher risk for future breakdown than gum bleeding. We also found that higher levels of neutrophils were linked to the presence of harmful bacteria that cause periodontitis. These findings suggest that measuring oral neutrophils could be a more reliable way to diagnose and monitor periodontitis, helping dentists identify severe cases earlier and treat patients more effectively. This method could improve how we understand and manage gum disease, leading to better patient‐centered outcomes.

## 1 INTRODUCTION

It has become increasingly evident that the negative effects of periodontitis reach far beyond the oral cavity; from diabetes mellitus to cardiovascular diseases and mental health disorders, an alarming number of associations have been, and are continuing to be, identified between periodontal inflammation and debilitating systemic inflammatory diseases.^1^, ^2^, ^3^, ^4^, ^5^ As periodontitis progresses, treatment outcomes, including tooth survival, are significantly reduced. This underlines the need for precise and reliable methods that facilitate screening and early diagnosis in attempt to improve overall prognoses for patients with periodontitis.

The current classification scheme for periodontitis^6^ was designed with this in mind. Diagnostic efforts have improved with the utilization of biologic information to estimate the potential systemic impact of the disease, and vice versa.^6^, ^7^, ^8^ In conjunction with clinical and radiographic parameters, the presence of systemic risk factors, termed grade modifiers, are considered to establish evidence‐based diagnoses. In doing so, clinicians may identify patients more accurately who appear to be increasingly susceptible to rapid periodontal breakdown, such as those with hyperactive immunoinflammatory responses,^9^, ^10^, ^11^ and implement appropriate interventions based on priority.^8^ Moreover, clinical and radiographic data mostly provide reliable information for previous disease activity only; whereas it has been hypothesized that biologic factors may lend themselves to quantify active disease more precisely.^12^, ^13^ Specific risk factors for periodontitis, including cigarette smoking and poor glycemic control, are currently accepted grade modifiers; however, additional oral and systemic inflammatory biomarkers have been under investigation for their possible use as prognostic indicators for periodontitis.^13^, ^14^, ^15^

Oral polymorphonuclear neutrophils (oPMN), and more specifically their various phenotypes, are potentially among the most important biological factors which are becoming increasingly understood and which may be able to predict disease prognosis and control.^14^, ^16^, ^17^, ^18^, ^19^, ^20^ oPMN are the primary mediators of oral immunity, and various oPMN subsets exist, differentiated by surface marker expression, in health and disease.^12^, ^21^ Previous studies have identified an interplay between the activation states of oral and blood neutrophils in gingivitis^22^ and, more importantly, have shown that the level of salivary oPMN correlates with periodontitis severity and presence of active oral inflammation.^18^, ^23^, ^24^, ^25^ To date, sophisticated point‐of‐care tests (POCT) have been developed and fine‐tuned for the detection of oral inflammatory load (OIL) based on the levels of salivary oPMN, thereby providing a noninvasive chairside test which can be used to screen patients based on oPMN count.^14^, ^23^, ^25^, ^26^, ^27^, ^28^ Data obtained through these clinician‐ and patient‐friendly POCT for oPMN counts are completely objective, offering a significant advantage over traditional methods of clinical testing, such as probing depth (PD), bleeding on probing percentage (BOP%), and clinical attachment loss (CAL), which tend to be susceptible to a high degree of user error and reduced sensitivity, effectively making these measures somewhat more subjective than objective.^29^, ^30^, ^31^, ^32^, ^33^ Furthermore, obtaining clinical data requires a comprehensive periodontal exam which can be uncomfortable for patients and, although less important from the perspective of disease, is a time‐consuming procedure for clinicians.^34^

Despite these limitations, the identification of periodontal inflammation is based on the presence and degree of BOP (i.e., as the gold standard). BOP is the result of ulceration of the sulcular epithelium caused by manipulation of these tissues, normally by a periodontal probe, in the presence of pathogenic bacteria.^35^, ^36^, ^37^ While presence of BOP does not necessarily mean a priori that these tissues are *inflamed*, the absence of BOP does suggest periodontal stability.^29^, ^30^, ^32^ Nonetheless, the presence of BOP might be indicative of underlying inflammation; however, the relationship between BOP and oral inflammation is inconsistent and appears to rely on the microbial nature of the biofilm.^25^, ^38^, ^39^ oPMN accumulate within the gingivocrevicular fluid (GCF), alongside biofilms rich in periodontal pathogens including *Porphyromonas gingivalis* (*Pg*), *Treponema denticola* (*Td*), *Tannerella forsythia* (*Tf*), *Aggregatibacter actinomycetemcomitans* (*Aa*), and *Prevotella intermedia* (*Pi*), and tend to be associated with patients who are at risk of future disease progression.^37^, ^39^ In theory, given that both BOP% and oPMN level are clinical markers of oral inflammation, the levels of salivary oPMN should be somewhat proportionate to the presence of BOP% in terms of classifying inflammatory‐driven periodontal diseases and conditions. However, as previously suggested by Jiang et al., specific interactions exist between oPMN and periodontal pathogens and therefore should be reflected by correlations between the two,^39^ whereas BOP may not be directly related. Given that oPMN are at the forefront of oral immunity, we hypothesized that salivary oPMN levels are a more effective, easily obtainable, and less‐invasive biomarker of both oral inflammation and the presence of pathogenic species compared to BOP%.

The quantitation and characterization of oPMN as prognostic markers for periodontitis has significant potential; however, there are limited reports which have evaluated the overall clinical efficacy of this approach for staging and grading purposes. Likewise, associations between oPMN level and BOP% with the presence of putative periodontal pathogens within the GCF have not been studied comparatively in vivo. Therefore, the main objectives of this work were twofold: to investigate the prognostic utility of oPMN count, as compared with BOP%, as a clinical marker for inflammation in the presence of periodontitis with advanced stages (i.e., 2 and 3) and grades (i.e., B and C) and to elucidate potential associations between oPMN count and BOP% with the presence of pathogenic microbial species *Pg*, *Td*, *Tf*, *Aa*, and *Pi* in a clinical setting.

## 2 MATERIALS AND METHODS

### 2.1 Study design and population

All patients who were considered for inclusion in this study were referred to a dedicated treatment and investigative clinical program at Mount Sinai Hospital, Department of Dentistry (Toronto, Canada). Patients were either referred by general dentists, or periodontists, for assessment of advanced periodontitis which had previously shown *resistance* or *nonresponsiveness* to all conventional forms of standard periodontal therapy (SPT). These included oral hygiene instructions (OHI) with notable improvement in oral hygiene practices, regular intervals of nonsurgical therapy (i.e., scaling and root planing with or without local anesthetics every 3–4 months), administration of systemic and/or locally delivered antibiotics at areas of persistent periodontal breakdown, and surgical periodontal therapy (i.e., open flap debridement with or without osseous resection surgery and/or periodontal regeneration).

In addition to exhausting the above list of therapeutic interventions, for patients to be admitted into this clinical program, they must also have demonstrated four out of the five following features of ongoing and poorly responsive periodontitis:

1. Ongoing and/or unexplained persistent bleeding and pain during periodontal probing
2. Ongoing and/or unexplained loss of clinical attachment and increases in gingival recession
3. Persistent and/or unexplained increases in periodontal pockets despite adequate SPT
4. Recurrent periodontal abscesses
5. Twenty percent of teeth with Class 1–3 mobility.

A total of 73 subjects were identified to be in alignment with the above acceptance criteria to be considered for inclusion in this study (REB 30044, 29410) which met the requirements of *high‐risk* periodontitis (see above explanation). After initial identification of this high‐risk population, the following clinical inclusion and exclusion criteria were applied to minimize the risk for potentially confounding variables. Subjects with complete clinical records at initial assessment and those who were either fully or partially dentate (i.e., ≥20 teeth, excluding third molars) were included. Subjects with incomplete records, complete edentulism, peri‐implantitis, a history of host modulation therapy (HMT) prior to initial assessment, and severe systemic diseases attributing to variance in the parameters of interest (e.g., leukocyte and/or immunodeficiency disorders) were excluded.

The final study population consisted of 62 subjects (34 male, 28 female) aged 15–79 years old. Subjects were grouped into one of four categories based on their initial periodontal diagnosis: stage II periodontitis (S2P), stage III periodontitis (S3P), grade B periodontitis (GBP), and grade C periodontitis (GCP), as listed in Table 1.

**TABLE 1:** Demographic data, including clinical periodontal parameters and inflammatory markers.

Patient characteristic^a^	S2P (*n* = 19)	S3P (*n* = 43)	*p*	GBP (*n* = 34)	GCP (*n* = 28)	*p*
Age (years)	55.37 ± 10.87	49.09 ± 12.70	0.07	53.94 ± 10.89	47.46 ± 13.43	^*^
Sex						
Male	7	27	^***^	16	18	^*^
Female	12	16		18	10	
**Smoking status** ^a^						
Never smoker	14	28	^*^	19	23	^**^
Past smoker	5	12		14	3	
Current smoker	0	3		1	2	
**Periodontal parameters** ^b^						
No. of teeth	27.42 ± 2.01	27.88 ± 0.77	0.24	27.38 ± 1.95	27.72 ± 1.17	0.45
PD (mm)						
1–3	147.63 ± 14.50	129.67 ± 22.92	^**^	140.47 ± 18.87	128.75 ± 24.52	^*^
4–6	17.11 ± 13.64	32.95 ± 20.27	^**^	23.21 ± 15.60	34.04 ± 22.84	^*^
>6	0.00 ± 0.00	1.13 ± 1.51	^**^	0.50 ± 0.80	1.24 ± 1.98	0.06
CAL (mm)						
1–3	132.37 ± 25.25	112.42 ± 46.38	0.08	126.74 ± 32.27	108.57 ± 50.05	0.09
4–6	31.47 ± 23.75	43.58 ± 39.82	0.22	33.71 ± 27.15	47.36 ± 43.68	0.14
>6	0.00 ± 0.00	6.46 ± 6.62	^***^	2.63 ± 3.83	7.58 ± 8.63	^**^
**Inflammatory markers** ^c^						
oPMN (10^6^/mL)	0.77 (0.48, 1.42)	2.51 (1.10, 4.08)	***	0.80 (0.68, 1.24)	3.01 (1.10, 4.20)	***
BOP (%)	16 (10, 32)	34 (22, 52)	**	17 (12, 30)	39.5 (23, 52)	*

Abbreviations: BOP, bleeding on probing; CAL, clinical attachment loss; GBP, grade B periodontitis; GCP, grade C periodontitis; oPMN, oral polymorphonuclear neutrophil; PD, probing depth; S2P, stage II periodontitis; S3P, stage III periodontitis.

^a^ Demographic data derived from chi‐squared tests, represented as mean ± SD.

^b^ Periodontal parameters derived from one‐way analysis of variance (ANOVA) tests, represented as mean ± SD.

^c^ Inflammatory markers obtained from Mann–Whitney *U* tests (two‐sided), represented as median (95% CI).

* *p *< 0.05

** *p *< 0.01

*** *p *< 0.001.

### 2.2 Clinical examination

Complete medical, dental, social, and family histories were obtained. Each subject underwent a comprehensive periodontal examination using a periodontal probe* for assessment of PD, BOP%, and CAL. Radiographic examinations included a combination of bitewing, periapical, and/or panoramic radiographic images to assess periodontal bone loss. Periodontal diagnoses^6^, ^8^ were obtained retrospectively; patients without a recorded diagnosis were excluded to minimize the risk of selection bias.

### 2.3 Oral rinse sampling and oPMN quantification

An oral rinse assay was performed at initial examination to assess oPMN levels.^27^ Briefly, subjects prerinsed using tap water for 15 s, after which expectorated products were discarded. Following a waiting period of 2 min, a 30‐s subsequent rinse was performed using 10 mL of USP (United States Pharmacopoeia)‐grade water which was expectorated into a reaction cup. The purpose for this was to allow for the influx of oPMN into the oral cavity following the prerinse. The oral rinse samples were immediately fixed in a 15‐mL tube containing 2 mL of phosphate‐buffered saline (PBS) and paraformaldehyde. Laboratory quantification of oPMN levels was performed using a validated cell fixation method and staining using acridine orange. Counts of oPMN were obtained visually by a laboratory technician via the use of a hemocytometer and reported in units of millions per milliliter (10^6^/mL) (Table 2).

**TABLE 2:** Microbial parameters for study population.

Microbial markers^a^	Undetectable	Detectable	*p*
**oPMN (10^6^/mL)**			
Single species			
*Pg*	0.86 (0.71, 1.50)	2.97 (1.10, 4.08)	^**^
*Td*	2.20 (1.00, 3.53)	0.82 (0.70, 1.60)	0.07
*Tf*	1.43 (0.70, 2.92)	1.10 (0.80, 2.99)	0.58
*Aa*	1.10 (0.79, 1.78)	2.20 (0.68, 1.89)	0.72
*Pi*	1.00 (0.71, 1.78)	3.30 (0.83, 5.30)	^*^
Red complex			
1/3 sp.	1.04 (0.69, 2.35)	2.00 (0.65, 3.99)	0.27
2/3 sp.	1.04 (0.69, 2.35)	1.10 (0.74, 2.30)	0.97
3/3 sp.	1.04 (0.69, 2.35)	0.80 (0.46, 5.25)	0.60
**BOP (%)**			
Single species			
*Pg*	26.50 (16.0, 52.0)	22.00 (10.0, 41.0)	0.25
*Td*	34.50 (17.0, 52.0)	16.00 (10.0, 31.0)	^*^
*Tf*	21.50 (15.0, 48.0)	31.00 (13.0, 55.0)	0.51
*Aa*	30.00 (16.0, 54.0)	22.00 (13.0, 48.0)	0.32
*Pi*	22.50 (16.0, 38.0)	35.00 (7.0, 75.0)	0.59
Red complex			
1/3 sp.	28.00 (20.0, 43.0)	22.50 (13.0, 57.0)	0.89
2/3 sp.	28.00 (20.0, 43.0)	17.00 (12.0, 38.0)	0.36
3/3 sp.	28.00 (20.0, 43.0)	31.00 (0.0, 92.0)	0.76

Abbreviations: *Aa*, *Aggregatibacter actinomycetemcomitans*; BOP, bleeding on probing; oPMN, oral polymorphonuclear neutrophil; *Pg*, *Porphyromonas gingivalis*; *Pi*, *Prevotella intermedia*; sp., species; *Td*, *Treponema denticola*; *Tf*, *Tannerella forsythia*.

^a^ Microbial data obtained from Mann–Whitney *U* tests (two‐sided), represented as median (95% CI).

^*^ *p *< 0.05; ^**^ *p *< 0.01.

### 2.4 Biofilm isolation, DNA extraction, and polymerase chain reaction screening

Subgingival biofilms were collected as described previously.^40^ Briefly, four separate sterile endodontic paper points (size 30) were placed into the gingival sulci at randomized sites for a period of 30 s to harvest subgingival biofilm samples. The paper points were then placed in sand tubes† and stored at −20°C until future analysis. DNA extraction was carried out using an isolation kit‡ following the manufacturer's instructions. DNA was quantified using a spectrophotometer.§ A total of 10 ng of high quality DNA isolated from each plaque sample was used as a template for polymerase chain reaction (PCR) screening assay using a PCR reaction containing 2 µL of 10× PCR buffer, 0.4 µL of MgCl_2_ (final concentration 2 mM), 0.4 µL of deoxynucleotide triphosphate (dNTP) mix (final concentration 200 µM of each dNTP), 0.1 µL of DNA polymerase‖ (final concentration 2.5 units/reaction), 0.5 µL of each forward and reverse primer (final concentration 1 µM each), 5 µL of DNA template, and 11 µL nuclease‐free PCR water in a total of 20 µL PCR reaction. PCR cycling conditions included an initial heat activation step (95°C for 15 min), followed by three‐step cycling (×30 cycles) including denaturation at 94°C for 30 s, annealing 58°C, and extension at 72°C for 1 min. The last step after 30 cycles included a final extension at 72°C for 10 min. We used the following primer sequences to detect the periodontal pathogens *Pg*, *Td*, *Tf*, *Aa*, and *Pi* (one primer set per reaction):

*Pg*: 5′‐TGG TTT CAT GCA GCT TCT TT‐3/5‐TCG GCA CCT TCG TAA TTC TT‐3′
*Td*: 5′‐ CCT TGA ACA AAA ACC GGA AA‐3′/5′‐ GGG AAA AGC AGG AAG CAT AA‐3′
*Tf*: 5′‐ GGG TGA GTA ACG CGT ATG TAA CCT‐3′/5′‐ ACC CAT CCG CAA CCA ATA AA‐3′
*Aa*: 5′‐CTT ACC TAC TCT TGA CAT CCG AA‐3′/5′‐ ATG CAG CAC CTG TCT CAA AGC‐3′
*Pi*: 5′‐CGA ACC GTC AAG CAT AGG‐3′/5′‐AAC AGC CGC TTT TAG AAC ACA A‐3′

PCR products were combined with DNA loading dye and subjected to gel electrophoresis using 2% agarose gel. Amplicon bands were visualized using an imaging system to aid in analysis.¶

### 2.5 Statistical analyses

All data were analyzed using GraphPad Prism.# Descriptive statistics were used to assess patient characteristics including age, smoking history, and clinical periodontal parameters, as described in Table 1. Chi‐squared tests (two‐sided) were used for descriptive analyses of categorical data including gender differences (i.e., the proportions of males to females in Groups S2P, S3P, GBP, and GCP) and smoking status (i.e., the proportions of never, past, and current smokers in Groups S2P, S3P, GBP, and GCP) as well as for assessment of the distribution of *Pg*, *Td*, *Tf*, *Aa*, *Pi*, and combinations of red‐complex species across diagnostic Groups S2P, S3P, GBP, and GCP. (Supplementary Figures 1 and 2).

**FIGURE 1:**
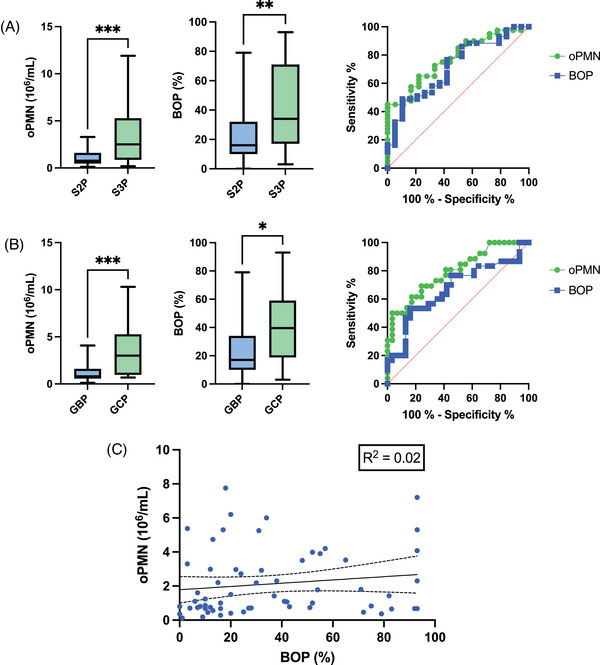
Elevated oPMN count correlates with increasing periodontitis stage and grade. Mann–Whitney tests (two‐tailed) yielded significant differences between oPMN levels and BOP for (A) S2P versus S3P (^***^ *p* < 0.001, ^**^ *p* < 0.01) and (B) GBP versus GCP (^***^ *p* < 0.001, ^*^ *p* < 0.05). ROC curves for stage (top right) and grade (middle right) were constructed to evaluate diagnostic accuracies of oPMN count (green) and BOP% (blue) (Table 3). (C) Simple linear regression indicates extremely weak correlation between oPMN count and BOP%; 95% CI featured with line of best fit. BOP%, bleeding on probing percentage; GBP, grade B periodontitis; GCP, grade C periodontitis; oPMN, oral polymorphonuclear neutrophil; ROC, receiver operating characteristic; S2P, stage II periodontitis; S3P, stage III periodontitis.

oPMN levels and BOP% were used as markers of inflammation and correlated with Groups S2P, S3P, GBP, and GCP (Table 1) as well as the presence of pathogenic bacteria *Pg*, *Td*, *Tf*, *Aa*, *Pi*, and a combination of one, two, or three red‐complex species (Table 2). Given our data were not normally distributed, Mann–Whitney *U* tests (two‐tailed) were used to evaluate associations between oPMN level and BOP% with S2P, S3P, GBP, and GCP and the presence of microbial pathogens, expressed as median with 95% CI. A Pearson test was performed to examine the correlations between oPMN count and BOP% (Figure 1C).

Receiver operating characteristic (ROC) curves were constructed using the Wilson–Brown method.^41^ Accuracy was determined based on the area under the curve (AUC) with 95% CI and standard error reported. Sensitivity (Se), specificity (Sp), and likelihood ratios (LR) were calculated to evaluate the efficacy of oPMN levels and BOP% as diagnostic (Table 3) and microbial biomarkers (Table 4). Comparisons between the ROC curves for statistically significant differences in the diagnostic and microbial parameters for oPMN count and BOP% were assessed using the Hanley–McNeil method.^42^ The ROUT method^43^ was applied at 1% aggressivity level to identify and eliminate outliers within all diagnostic groups to minimize skewedness. A level of significance *α* = 0.05 was used for all statistical analyses; *p* values <0.05 were reported as statistically significant.

**TABLE 3:** Diagnostic value of oPMN versus BOP as biomarkers for periodontitis stage and grade.

Diagnostic markers^a^, ^b^	Cutoff	Se	Sp	LR	AUC (95% CI)	*p*
oPMN (10^6^/mL)						
Stage	>1.43	65.00	77.78	2.93	0.776 (0.656, 0.897)	^***^
Grade	>1.43	69.23	75.86	2.87	0.792 (0.675, 0.910)	^***^
BOP (%)						
Stage	>16.50	76.74	57.89	1.82	0.715 (0.580, 0.851)	^**^
Grade	>22.50	70.00	58.06	1.67	0.670 (0.531, 0.810)	^*^

Differences in ROC curves^a^, ^c^	AUC (SE)	*z*‐score	*p*
Stage			
oPMN (10^6^/mL)	0.776 (0.061)	0.661	0.51
BOP (%)	0.715 (0.069)		
Grade			
oPMN (10^6^/mL)	0.792 (0.059)	1.32	0.18
BOP (%)	0.670 (0.071)		

Abbreviations: AUC, area under curve; BOP, bleeding on probing; LR, likelihood ratio; oPMN, oral polymorphonuclear neutrophil; ROC, receiver operating characteristic; Se, sensitivity; SE, standard error; Sp, specificity.

^a^ Prognostic values obtained from Mann–Whitney *U* tests (two‐sided), represented as median (95% CI).

^b^ Optimal cutoff points were determined by the Index of Union (IU) method.

^c^ ROC curves for oPMN and BOP were compared using the Hanley–McNeil method.

* *p *< 0.05.

** *p *< 0.01.

*** *p *< 0.001.

**TABLE 4:** Diagnostic value of oPMN versus BOP as biomarkers for presence of *Pg*, *Td*, *Tf*, *Aa*, *Pi*, or a combination of one, two, or three red‐complex species, respectively.

Microbial markers^a^, ^b^	Cutoff	Se	Sp	LR	AUC (95% CI)	*p*
**oPMN (10^6^/mL)**						
Single species						
*Pg*	>1.47	64.00	68.97	2.06	0.704 (0.562, 0.846)	^*^
*Td*	<1.17	62.50	61.29	1.62	0.645 (0.498, 0.793)	0.07
*Tf*	>1.05	56.00	45.16	1.02	0.545 (0.392, 0.697)	0.57
*Aa*	>1.33	52.63	54.29	1.15	0.531 (0.355, 0.707)	0.71
*Pi*	>2.00	57.89	70.27	1.95	0.708 (0.562, 0.855)	^*^
Red complex						
1/3 sp.	>1.60	55.56	59.09	1.36	0.577 (0.389, 0.765)	0.41
2/3 sp.	>1.09	53.33	54.55	1.17	0.532 (0.344, 0.720)	0.74
3/3 sp.	>1.25	42.86	54.55	0.94	0.555 (0.296, 0.814)	0.66
**BOP (%)**						
Single species						
*Pg*	<22.50	52.00	55.88	1.18	0.588 (0.438, 0.739)	0.25
*Td*	<25.50	65.22	58.33	1.57	0.654 (0.513, 0.795)	^*^
*Tf*	>27.00	56.00	55.56	1.26	0.550 (0.403, 0.697)	0.51
*Aa*	<24.00	57.14	57.5	1.35	0.578 (0.429, 0.726)	0.32
*Pi*	>31.50	57.89	59.52	1.43	0.545 (0.369, 0.719)	0.58
Red complex						
1/3 sp.	<27.50	55.00	52.00	1.15	0.524 (0.346, 0.702)	0.78
2/3 sp.	<25.00	52.94	56.00	1.20	0.599 (0.421, 0.776)	0.28
3/3 sp.	>29.00	57.14	52.00	1.19	0.517 (0.248, 0.786)	0.89

Differences in ROC curves^a^, ^c^	AUC (SE)	*z*‐score	*p*
*Pg*			
oPMN (10^6^/mL)	0.704 (0.073)	1.097	0.27
BOP (%)	0.588 (0.077)		
*Pi*			
oPMN (10^6^/mL)	0.708 (0.075)	1.410	0.16
BOP (%)	0.545 (0.089)		
*Td*			
oPMN (10^6^/mL)	0.645 (0.072)	0.084	0.93
BOP (%)	0.654 (0.075)		

Abbreviations: *Aa*, *Aggregatibacter actinomycetemcomitans*; AUC, area under curve; BOP, bleeding on probing; LR, likelihood ratio; oPMN, oral polymorphonuclear neutrophil; *Pg*, *Porphyromonas gingivalis*; *Pi*, *Prevotella intermedia*; ROC, receiver operating characteristic; Se, sensitivity; SE, standard error; Sp, specificity; sp., species; *Td*, *Treponema denticola*; *Tf*, *Tannerella forsythia*.

^a^ Prognostic values obtained from Mann–Whitney *U* tests (two‐sided), represented as median (95% CI).

^b^ Optimal cutoff points were determined by the Index of Union (IU) method.

^c^ ROC curves for oPMN and BOP were compared using the Hanley–McNeil method.

* *p *< 0.05

## 3 RESULTS

Demographic data for the study population are listed in Table 1. Significant differences in age were found between Groups GBP and GCP (*p *< 0.05), where GCP demonstrated the youngest portion of subjects overall (47.46 ± 13.43 years old). A greater ratio of male to female subjects was observed for Group S3P versus S2P (*p *< 0.001) and Group GCP versus GBP (*p *< 0.05). Likewise, the proportions of never, past, and current smokers were significantly different between S2P versus S3P (*p *< 0.05) and GBP versus GCP (*p *< 0.01), where a large proportion of subjects were identified as previous or never smokers.

In terms of periodontal parameters, an expected increase was found between both S2P/S3P and GBP/GCP in the mean number of sites with PD 1–3 mm (*p *< 0.01, *p *< 0.05), PD 4–6 mm (*p *< 0.05, *p *< 0.01), and CAL > 6 mm (*p *< 0.001, *p *< 0.01), respectively. Also, an increase in the mean number of sites with PD > 6 mm was found between S2P/S3P only (*p *< 0.01), which is consistent with the current diagnostic scheme.^8^ Interestingly, we did not find a statistically significant difference in the number of teeth present at initial examination between the Groups S2P/S3P, nor GBP/GCP.

### 3.1 Diagnostic marker: Elevated oPMN levels were associated with increased stage and grade of periodontitis

A significant positive association was found in the mean levels of both inflammatory markers oPMN level and BOP% between Groups S2P/S3P (*p *< 0.001, *p *< 0.01) and GBP/GCP (*p *< 0.001, *p *< 0.05), respectively (Figure 1A,B). A nearly four‐fold positive correlation in median oPMN level was found between GCP versus GBP, and a three‐fold increase in median oPMN level was noted between S3P versus S2P, whereas BOP% showed a two‐fold increase in median for the S2P/S3P and GBP/GCP comparisons. Interestingly, an extremely weak correlation between oPMN level and BOP% was identified by simple linear regression analysis (*R* ^2^ = 0.02) (Figure 1C).

ROC curves were constructed to assess the diagnostic values of oPMN level and BOP% in differentiating between advanced levels of disease severity (i.e., S3P vs. S2P) and rate of disease progression (i.e., GCP vs. GBP). As listed in Table 3, oPMN level demonstrated the greatest accuracy for differentiating GCP from GBP (AUC = 0.792, *p *< 0.001). This was also reflected by superior Se, Sp, and predictive values for oPMN count in differentiating between periodontitis grade, as compared to BOP%, based on ROC curve differences between GCP and GBP. In terms of differentiating S3P from S2P, both oPMN level and BOP% showed significance (*p *< 0.001, *p *< 0.01), with no significant differences in their ROC curves (*p* > 0.05); although, LR for oPMN level were greater than those for BOP% in both stage and grade.

### 3.2 Microbial marker: Elevated oPMN levels were associated with increased presence of pathogenic bacteria

**FIGURE 2:**
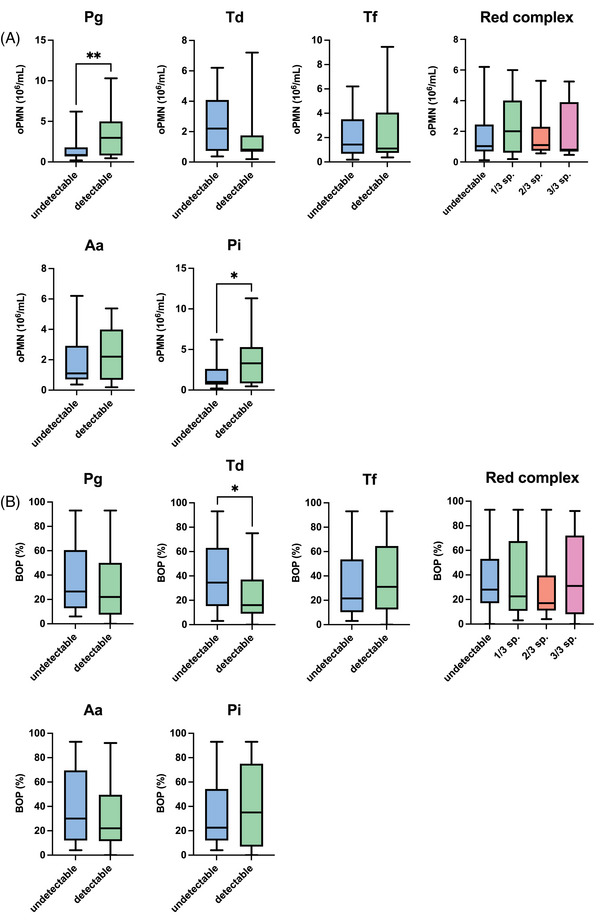
Elevated oral neutrophil count correlates with prevalence of specific oral pathogens in subgingival biofilm. Mann–Whitney *U* tests (two‐tailed) were used for monospecies analyses and one‐way analysis of variance for red‐complex combinations. (A) Increased oPMN level was associated with presence of *Pg* and *Pi* (^*^ *p* < 0.01), but not red complex. (B) BOP was inversely associated with presence of *Td* (^*^ *p* < 0.01). *Aa*, *Aggregatibacter actinomycetemcomitans*; BOP, bleeding on probing; oPMN, oral polymorphonuclear neutrophil; *Pg*, *Porphyromonas gingivalis*; *Pi*, *Prevotella intermedia*; sp., species; *Td*, *Treponema denticola*; *Tf*, *Tannerella forsythia*.

As illustrated in Figure 2, oPMN levels were associated with a significantly higher prevalence of *Pg* and *Pi* in comparison to BOP% (*p* < 0.01) (Table 2). Interestingly, BOP% demonstrated an inverse correlation with the presence of *Td* (*p* < 0.05). No significant differences were observed between oPMN levels and BOP% in the prevalence of a combination of one, two, or three red‐complex species (*p* > 0.05).

To assess the diagnostic value of oPMN level and BOP% as microbial biomarkers, ROC curves were constructed for each individual pathogen, along with the presence of one, two, or three red‐complex species (Supplementary Figure 1). The diagnostic values for oPMN level and BOP% as microbial biomarkers are listed in Table 4. Like the trends noted above, the diagnostic value of oPMN level was significantly associated with the increased presence of *Pg* and *Pi* (*p* < 0.05), while BOP% was found to be significantly associated with a lower prevalence of *Td* (*p* < 0.05). Nonetheless, no significant differences were observed in the diagnostic values between oPMN level and BOP% when comparing the ROC values for *Pg*, *Pi*, and *Td* (*p *> 0.05).

### 3.3 Red‐complex bacteria were associated with increased stage of periodontitis

The relative distributions of S2P, S3P, GBP, and GCP within the microbial data were also assessed. Results revealed a higher proportion and greater difference between the prevalence of red‐complex species in S3P and S2P (*p *< 0.05) (Figure 3; Supplemental data). Interestingly, the remaining microbial data did not demonstrate significant differences in periodontitis stage and grade. In fact, as illustrated in Supplementary Figure 2, most microbial pathogens were undetectable for each diagnostic group, including S3P and GCP.

## 4 DISCUSSION

This study was conducted to evaluate the prognostic value of oPMN level compared to BOP% to evaluate the usefulness for more biologically driven assessment of periodontitis stage and grade. Although we have previously carried out a large multicenter clinical study on a similar topic,^25^ the population reported herein was considerably unique. In this work, we focused on a *high risk* or *treatment‐resistant* subset of patients with periodontitis, as described above. Additionally, we aimed to compare the potential associations between oPMN and BOP% with the presence of periodontal pathogens *Pg*, *Td*, *Tf*, *Aa*, and *Pi* in the GCF. Given that oPMN are readily available in the GCF and saliva, with significant advancements in isolation and quantification assays,^18^, ^27^ assessment of the levels and phenotypes of oPMN have garnered great interest as a potential prognostic biomarker for periodontitis.^14^ Furthermore, with the lack of reliability of BOP% as an oral inflammatory marker, we hypothesized that the use of oPMN level may be a more reliable POCT to assess for OIL, as described previously, in comparison to BOP%.^23^, ^28^ Our results support our hypothesis in that oPMN boasted a significant advantage over BOP% as an inflammatory marker, which was reflected further by changes to the microbiome in advanced stages and grades of periodontitis. To our knowledge, this is the only clinical study to date which corroborates associations between oPMN level, periodontal diagnosis, and microbial data within a high‐risk periodontitis population.

### 4.1 Elevated oPMN levels can differentiate periodontitis stage 2 versus 3 and grade B versus C

Interestingly, although oPMN level and BOP% are both clinical markers of inflammation, we found an extremely weak correlation between them (*R* ^2^ = 0.02) (Figure 1C). Elevated levels of oPMN were positively associated with advanced stage and grade of periodontitis. Specifically, oPMN count outperformed BOP% in its ability to identify patients with advanced periodontal disease and risk of progression (i.e., S3P vs. S2P and GCP vs. GBP) (Figure 1A,B). These findings are consistent with those of previous studies,^18^, ^24^, ^27^ which demonstrated a positive correlation between oPMN levels and periodontitis disease severity (i.e., periodontitis stage) and risk for disease progression (i.e., periodontitis grade).^25^ Furthermore, our results indicated that oPMN levels could also be used to reliably differentiate between periodontitis grades B versus C, suggesting that oPMN correlates with increased rates of periodontal destruction and a higher risk for future disease progression.

Most of our subjects were not current smokers (Table 1), thereby minimizing the potential effects of this grade‐modifying confounder. The diabetic status for each subject (e.g., glycosylated hemoglobin [HbA1c] %) and systemic levels of high‐sensitivity C‐reactive protein (hs‐CRP mg/L) were not determined for all subjects and therefore could not be included in our analyses. Given the retrospective nature of this study, we suggest oPMN levels should be considered as a risk indicator for periodontitis and to differentiate grade C versus grade B; however, future prospective clinical studies are required to elevate oPMN level to the category of “risk factor.”

To our knowledge, this is the first study which estimates and quantifies the clinical value of oPMN level to differentiate between periodontitis stages 2 and 3 and grades B and C in a high‐risk patient population. As listed in Table 3, the optimal oPMN cutoff value was determined to be 1.43 × 10^6^/mL for both stage and grade as per the Index of Union (IU) method.^44^ oPMN levels greater than the optimal threshold indicated the greatest LR for S2P versus S3P (2.87) and GBP versus GCP (2.87). In comparison, BOP% cutoff values were inconsistent, which may be a consequence of increased variability in BOP measurements due to challenges in obtaining consistency in probing forces and gingival tissue phenotype (e.g., tissue fragility) differences between patients. Nonetheless, the LR for S3P versus S2P and GCP versus GBP were weaker for BOP% than oPMN count. It is important to acknowledge, however, that the magnitude of LR+ results in a small to moderate increase in the probability of predicting GCP and S3P using oPMN levels alone.

Se and Sp values were also determined for oPMN level and BOP% (Table 3). Based on our results, oPMN count demonstrated a higher Sp for advanced stage and grade of periodontitis than BOP%. This suggests oPMN levels greater than the cutoff value of 1.43 × 10^6^/mL may be used to differentiate S3P versus S2P and GCP versus GBP. Given that the summation of Se and Sp for oPMN levels was <1.5,^45^ our results suggest that an oPMN level >1.43 × 10^6^/mL is a potentially useful test to differentiate S3P and GCP from S2P and GBP; however, this requires further investigation. Previous studies have suggested that BOP% lacks Sp, such that the absence of BOP indicates periodontal stability, whereas the presence of BOP does not reliably indicate periodontal inflammation.^32^ We found similar results, in that BOP% was less specific for periodontitis stage and grade.

### 4.2 Elevated oPMN levels are associated with the presence of *Pg* and *Pi* in the GCF

In addition to periodontal stage and grade, elevated levels of salivary oPMN were associated with the prevalence of *Pg* and *Pi* in the GCF. The prognostic values for oPMN level associated with *Pg* and *Pi* were determined (Table 4). Interestingly, the optimal oPMN cutoff value for *Pg* was determined to be 1.47 × 10^6^/mL, which was very similar to that for differentiating between stage and grade, while it was determined to be 2.00 × 10^6^/mL for *Pi*. We determined that oPMN level exhibits a higher Sp for increased prevalence of *Pg* and *Pi*; hence, these results may suggest that an oPMN level >1.47 × 10^6^/mL could be potentially useful to identify periodontal pockets with high concentrations of *Pg* and *Pi* (e.g., promoting targeted antimicrobial therapy in recurrent periodontal pockets); however, in comparison to BOP% the differences observed were not statistically significant.

One of the other interesting findings relates to the apparent divergence between changes in BOP% and oPMN levels in some instances. We suggest that this could mean that some sites with BOP might not actually be inflamed and not at risk for disease advancement. This idea is not new. Indeed, as shown previously, BOP% was shown to be a poor marker for active inflammation‐mediated destruction of periodontal tissue.^30^ Further along these lines, it was shown by Lang et al. that the *absence* of BOP was an excellent indicator of periodontal stability (i.e., no loss of attachment).^29^ Our data seem to suggest that certain bacteria (i.e., *Pg* and *Pi*), when present, can produce increases in BOP as well as elevations in oPMN levels. Perhaps this is reflective of the presence of sites with BOP that are inflamed and at risk of advancing. Although this is speculative, it is possible that some microbial species in the plaque/biofilm, such as *Td*, play a predominant role in causing epithelial disaggregation, but perhaps not as much a role in upregulation of inflammation.^46^, ^47^ In such instances, BOP would still be expected to occur, as a periodontal probe would penetrate the epithelial layers and disrupt the underlying connective tissue, including injury of blood vessels leading to bleeding. Perhaps these bacteria (e.g., *Td*) could facilitate invasion of the more inflammogenic bacteria, such as *Pg* and *Pi*, and further in this regard, sites infected with these bacterial types would not only have BOP but would also likely contribute to increases in the levels of oPMN.

### 4.3 Study limitations

Given the retrospective nature of this study, a temporal relationship cannot be drawn from our results to suggest whether oPMN level can be considered a risk factor which accounts for the transition from S2P to S3P, nor GBP to GCP; however, prospective clinical trials are currently underway to investigate this. Additionally, with respect to the demographic data, we did not find a statistically significant difference between S2P and S3P in terms of tooth loss (Table 1), although this does not necessarily align with the staging table for periodontitis.^8^ This might be attributed to tooth loss due to nonperiodontitis causes;^48^ however, the clinical parameters listed in Table 1 otherwise support the diagnoses which were retrospectively deduced.

Additionally, this study primarily focused on identifying the possible relationships between individual bacteria and inflammatory markers oPMN level and BOP% in attempt to provide an explanation as to whether any of these species plays a particular role in the onset and propagation of oral inflammation; however, this does not necessarily bode well with the fact that subgingival biofilms are increasingly sophisticated and complex, featuring a wide variety of microbes.^37^, ^39^ Future work may be directed toward studying the associations between synergistic clusters of mixed biofilm complexes, in alignment with the individual species studied here, and oPMN levels to better elucidate the roles of *Pg* and *Pi* in terms of oral inflammation and highlight trends in microbial composition which may affect OIL.

Our results showed a relatively high degree of variance, which might be attributed to randomized sampling at sites with varying levels of periodontal inflammation. Furthermore, the clinical testing was not performed by the same individual for all patients; rather, these tests were performed by postgraduate residents in periodontology at the University of Toronto during their rotations at the Mount Sinai Hospital Department of Dentistry, which could incorporate moderate interoperator variability into our results.

## 5 CONCLUSION

We found a correlation between oPMN level and advanced stage and grade of periodontitis, as well as with the presence of *Pg* and *Pi* in the GCF, whereas BOP% was associated with advanced stage and grade of periodontitis only. It was determined that above the threshold of an oPMN level of 1.43 × 10^6^/mL, patients with periodontitis were more likely to be diagnosed with S3P and GCP than S2P and GBP, respectively. These findings suggest that oPMN level can potentially serve as a diagnostic risk marker for periodontitis; as such its inclusion in the grading table^8^ should be considered. Although this requires additional study, this investigation provides a platform that brings us one step closer to being able to use early screening efforts which will allow us to triage patients with periodontitis more accurately, thus supporting the use of targeted periodontal therapies.

## AUTHOR INFORMATION

Braedan R. J. Prete is a postgraduate resident in periodontology at the University of Toronto, Canada. Abdelahhad Barbour is a research associate in oral microbiome, biofilms, and probiotics research. Chunxiang Sun is a lab manager. Michael B. Goldberg, Howard C. Tenenbaum, and Michael Glogauer are professors in periodontology at the University of Toronto, Canada. Michael B. Goldberg is the dentist‐in‐chief and head of periodontics at Sinai Health, Toronto, Canada. Michael Glogauer is the head of dental oncology at Princess Margaret Cancer Centre, Toronto, Canada.

## AUTHOR CONTRIBUTIONS

**Abdelahhad Barbour, Howard C. Tenenbaum, Michael B. Goldberg, Michael Glogauer**: Conception and project design. **Abdelahhad Barbour, Chunxiang Sun**: Sample collection and data acquisition. **Braedan R. J. Prete, Abdelahhad Barbour, Michael Glogauer**: Data analysis and interpretation. **Braedan R. J. Prete, Abdelahhad Barbour, Michael Glogauer**: Initial drafts. **Braedan R. J. Prete, Abdelahhad Barbour, Michael B. Goldberg, Howard C. Tenenbaum**: Critical revision of the manuscript. **Howard C. Tenenbaum, Michael B. Goldberg, Michael Glogauer**: Provision of clinical and laboratory resources for the study. All authors contributed to the realization of this paper and approved the manuscript prior to submission.

## CONFLICT OF INTEREST STATEMENT

Michael Glogauer is the inventor of, and holds a patent for, PerioMonitor, a chairside point‐of‐care test used to monitor oral inflammatory load. PerioMonitor is currently undergoing a human clinical trial sponsored by Oral Science International Inc. (NCT05886855). The authors have no additional conflicts of interest to declare with respect to the research, authorship, and/or publication of this article.

## Supporting information



ROC curves for microbial monospecies *Pg*, *Td*, *Tf*, *Aa*, and *Pi* and combinations of one, two, or three red‐complex species presence to determine diagnostic accuracy of oPMN (green) and BOP (blue) as microbial biomarkers. All diagnostic values can be found in Table 4.

Frequency distributions of S2P, S3P, GBP, and GCP patients for microbial biomarkers *Pg*, *Td*, *Tf*, *Aa*, and *Pi* expressed as (A) total subjects and (B) proportions. The majority of microbial species were undetectable by quantitative polymerase chain reaction (qPCR), although no statistical differences were noted (*p* > 0.05).

Frequency distributions of S2P, S3P, GBP, and GCP patients for combinations of one, two, or three red‐complex species expressed as total subjects and proportions. Significant differences were observed between proportions of S3P versus S2P (*p* < 0.05); however, no further significant differences were observed.

## Data Availability

The data that support the findings of this study are available in the Supplementary Material of this article.

## Footnotes

* Florida Probe, Gainesville, Florida, USA.

† PowerBead; Qiagen, Hilden, Germany.

‡ DNeasy PowerSoil Pro Kit (cat. no./ID 47014); Qiagen, Hilden, Germany.

§ NanoDrop 2000 UV Visible Spectrophotometer; Thermo Scientific, Canada.

‖ HotStarTaq DNA Polymerase; Qiagen, Hilden, Germany.

¶ Odyssey XF Imaging System; LI‐COR Biosciences, USA.

# Prism 10 version 10.1.1; GraphPad Software LLC, Boston, Massachusetts, USA.
