# Supplementary figures and images for: Prognostic utility of oral neutrophil counts in high‐risk periodontitis: A retrospective study
Source: J Periodontol. 2025 Jul 8;96(12):1352–65. doi: 10.1002/jper.11360 (PMC12819364; doi:10.1002/jper.11360)

**Pg**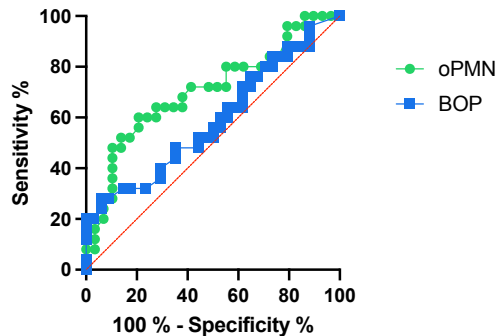**Td**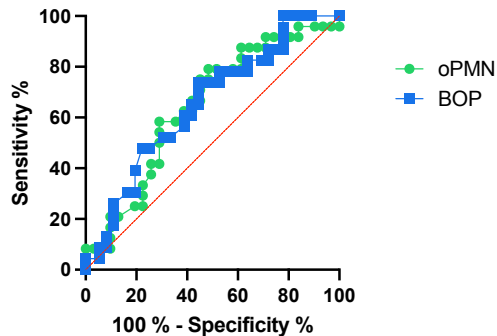**Tf**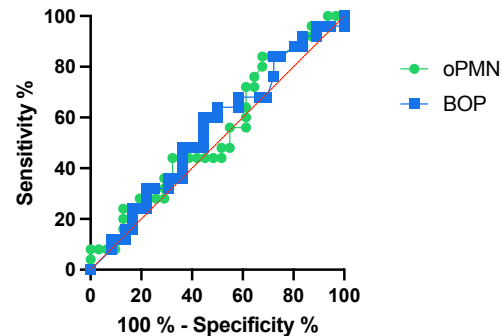**1/3 sp.**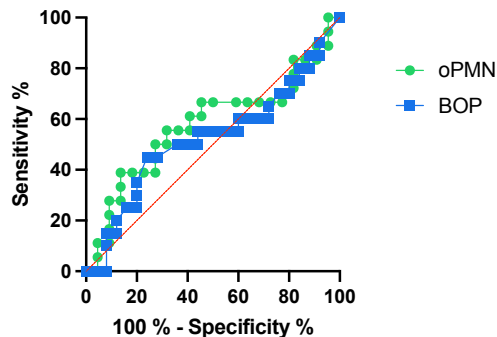**2/3 sp.**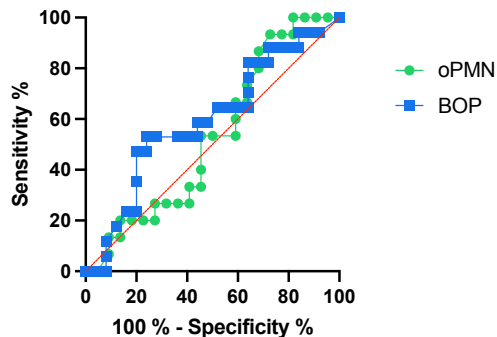**3/3 sp.**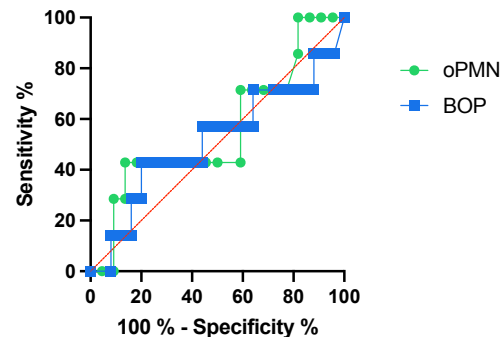**Aa**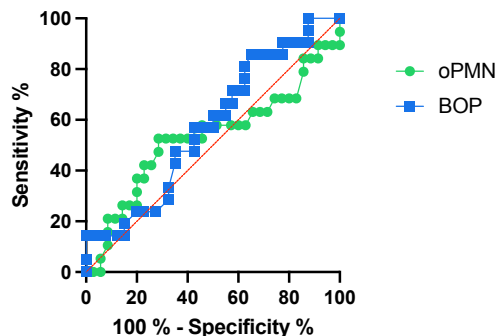**Pi**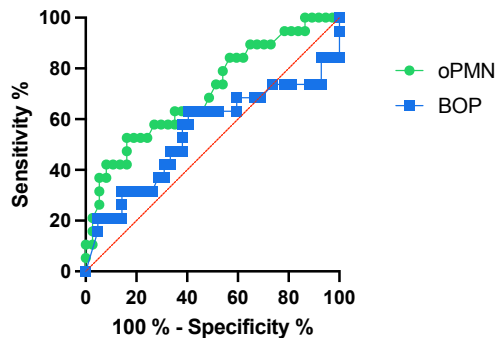

Supplement: Supplementary file 1 — ROC curves for microbial monospecies Pg, Td, Tf, Aa, and Pi and combinations of one, two, or three red‐complex species presence to determine diagnostic accuracy of oPMN (green) and BOP (blue) as microbial biomarkers. All diagnostic values can be found in Table 4. [file JPER-96-1352-s001.pdf]

(a)

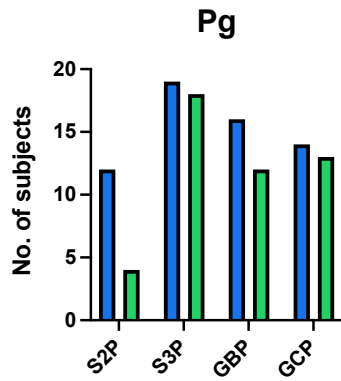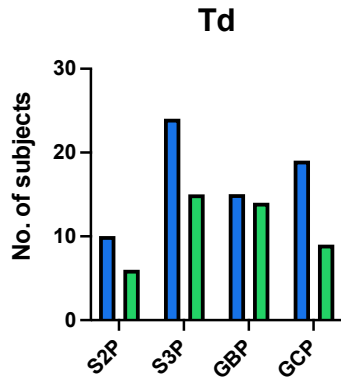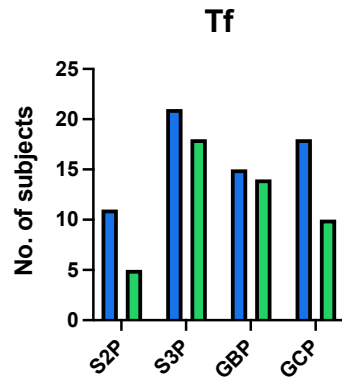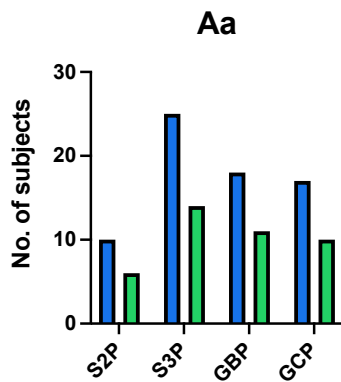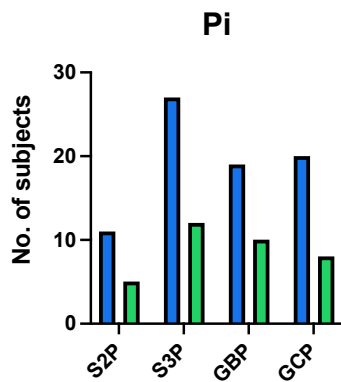

Undetectable  
Detectable

(b)

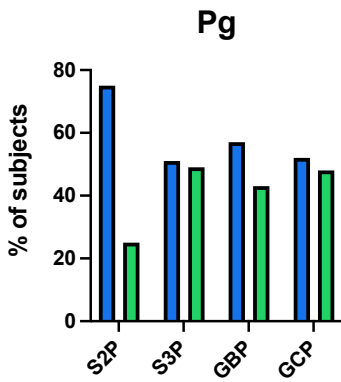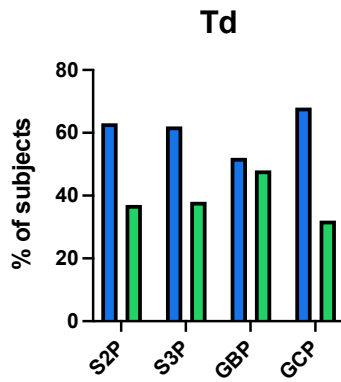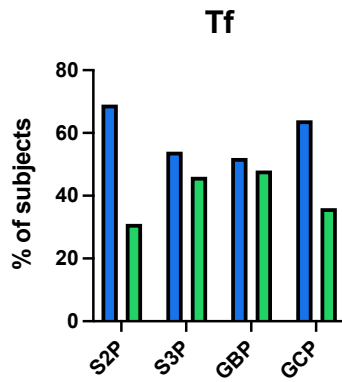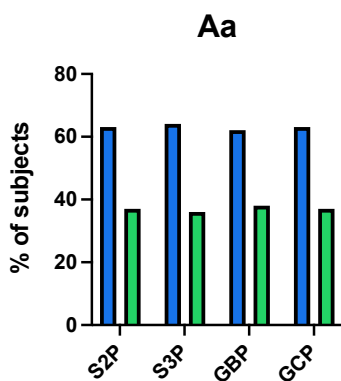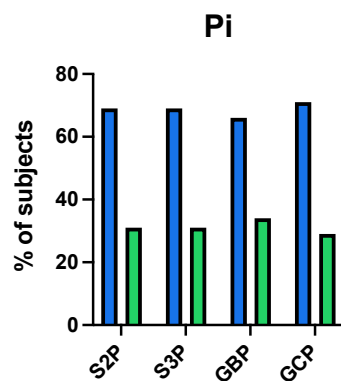

Undetectable  
Detectable

Supplement: Supplementary file 2 — Frequency distributions of S2P, S3P, GBP, and GCP patients for microbial biomarkers Pg, Td, Tf, Aa, and Pi expressed as (A) total subjects and (B) proportions. The majority of microbial species were undetectable by quantitative polymerase chain reaction (qPCR), although no statistical differences were noted (p > 0.05). [file JPER-96-1352-s002.pdf]

### Red complex

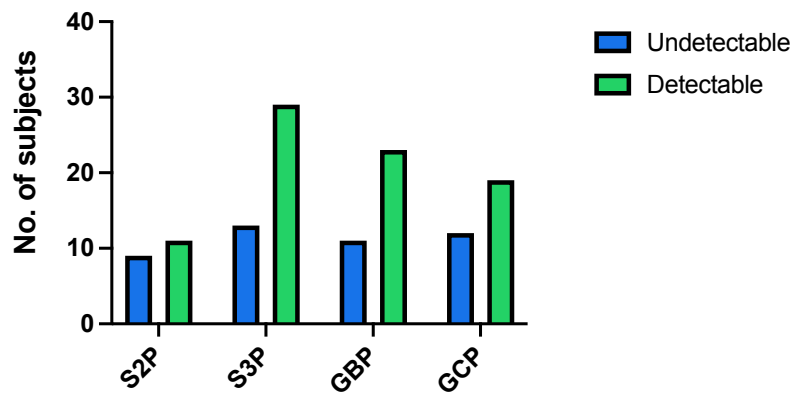

### Red complex

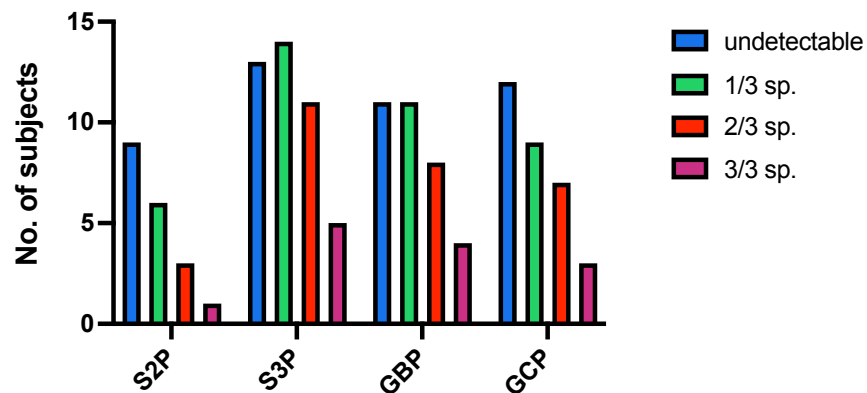

### Red complex

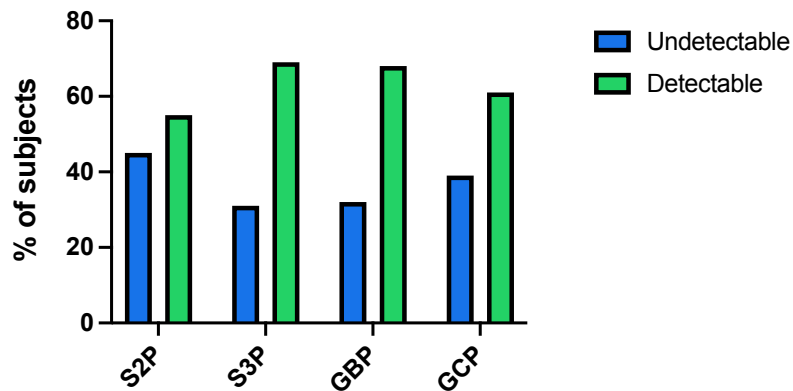

### Red complex

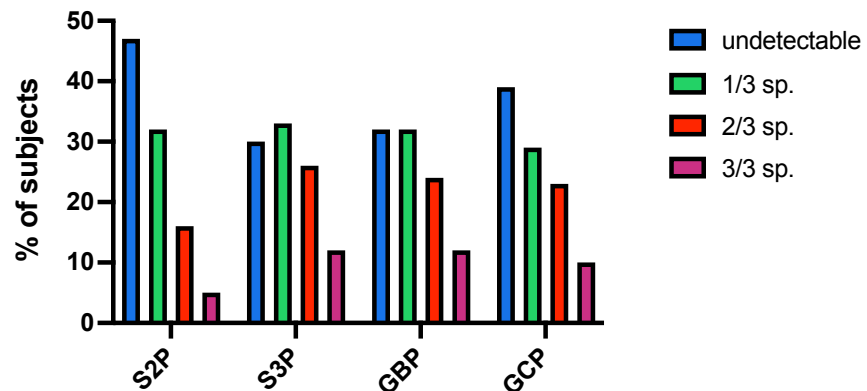

Supplement: Supplementary file 3 — Frequency distributions of S2P, S3P, GBP, and GCP patients for combinations of one, two, or three red‐complex species expressed as total subjects and proportions. Significant differences were observed between proportions of S3P versus S2P (p < 0.05); however, no further significant differences were observed. [file JPER-96-1352-s003.pdf]
